# Delayed surgical treatment for a traumatic bilateral cervical facet joint dislocation using a posterior-anterior approach: a case report

**DOI:** 10.1186/1752-1947-7-9

**Published:** 2013-01-09

**Authors:** Takashi Shimada, Seiji Ohtori, Gen Inoue, Junichi Nakamura, Izumi Nakada, Hiroshi Saiki, Song Ho Chang, Koui Kawamura, Kazuhisa Takahashi, Hiroshi Sugiyama

**Affiliations:** 1 Department of Orthopedic Surgery, Asahi General Hospital, i-1326, Asahi-shi, Chiba, 289-2511, Japan; 2 Department of Orthopedic Surgery, Graduate School of Medicine, Chiba University, 1-8-1 Inohana, Chuo-ku, Chiba, 260-8670, Japan

## Abstract

**Introduction:**

There have been few reports of patients with bilateral cervical facet dislocations that remain untreated for eight weeks or more. We report the case of a 76-year-old man with an old bilateral cervical facet joint dislocation fracture that was treated by posterior-anterior reduction and fixation.

**Case presentation:**

A 76-year-old Asian man was involved in a road traffic accident. He presented with neck pain and arm pain on his right side, but motor weakness and paralysis were not observed. He was treated conservatively; however, instability and spondylolisthesis at the C5 to C6 joint increased eight weeks after the injury. We performed a posterior-anterior reduction and fixation. After surgery, bony union was achieved, and his neck pain and arm pain disappeared.

**Conclusion:**

We recommend reduction and fixation surgery if a patient has an old bilateral facet joint dislocation fracture in the cervical spine.

## Introduction

Several authors have reported failures to correctly diagnose cervical spine injuries. The rate of misdiagnosis ranges from 5% to 20% [1-3]. Of missed spinal injuries, misdiagnosis of an injury to the cervical spine has been most frequently reported [3]. The use of computed tomography (CT) and magnetic resonance imaging (MRI) can improve the accuracy of cervical spine injury diagnosis. However, an analysis of the clinical records of 367 patients with cervical spine injuries revealed a diagnostic failure rate of 4.9% [4].

Treatment for acute cervical dislocation fracture includes conservative treatment and surgery. Whether conservative treatment is sufficient is controversial [5-7]. Recently, the incidence of surgical treatment to reduce dislocation and for fixation has increased compared with conservative treatment.

An injury is considered as ‘old’ when the interval between the accident and correct diagnosis is longer than three weeks [8]. There are several reports on the management of old dislocations of the subaxial cervical spine in the English literature [9-13]. Among these, only three articles were found in which the delayed treatment of bilateral cervical facet dislocations is described after a diagnostic delay of more than one month [9-11].

Here, we report a case of a 76-year-old man with an old bilateral cervical facet joint dislocation fracture that was treated by posterior-anterior reduction and fixation.

## Case presentation

A 76-year-old Asian man was involved in a road traffic accident. He presented with neck and arm pain on his right side, but motor weakness and paralysis were not observed. His arm pain corresponded to the right C6 and C7 dermatomes.

**Figure 1:**
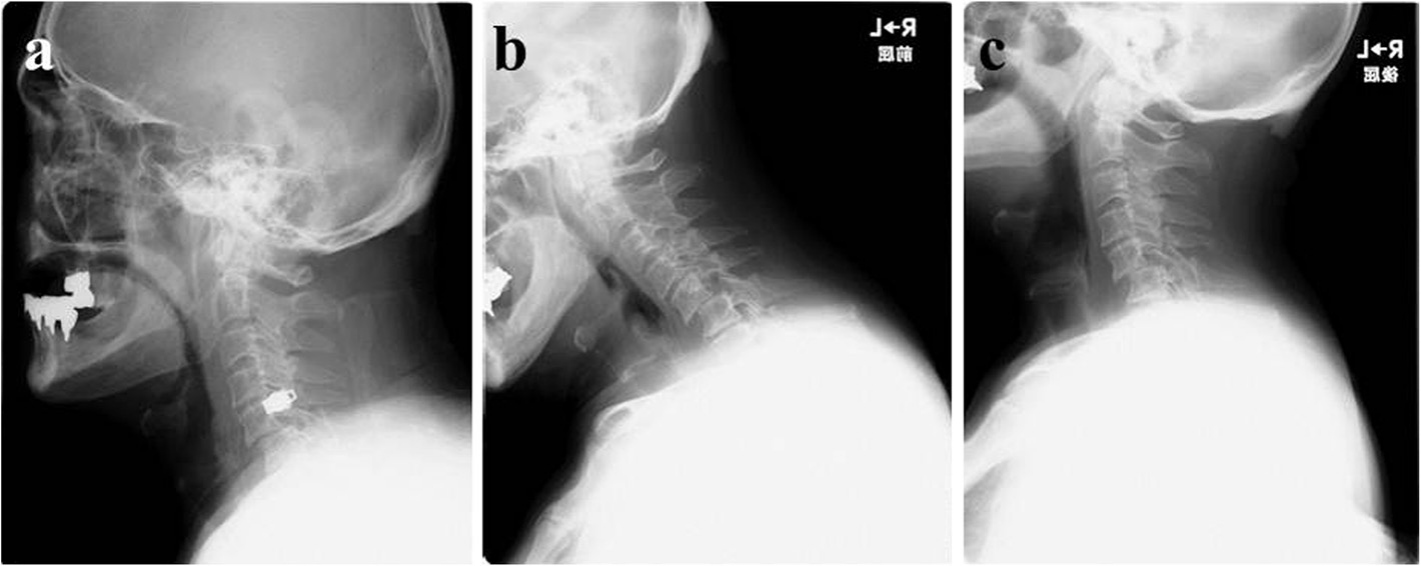
**Plain X-ray film images showing slight spondylolisthesis of C5 immediately after a traffic accident. (a)** The extent of the spondylolisthesis increased over the following eight weeks. **(b)** Flexion position; **(c)** extension position.

**Figure 2:**
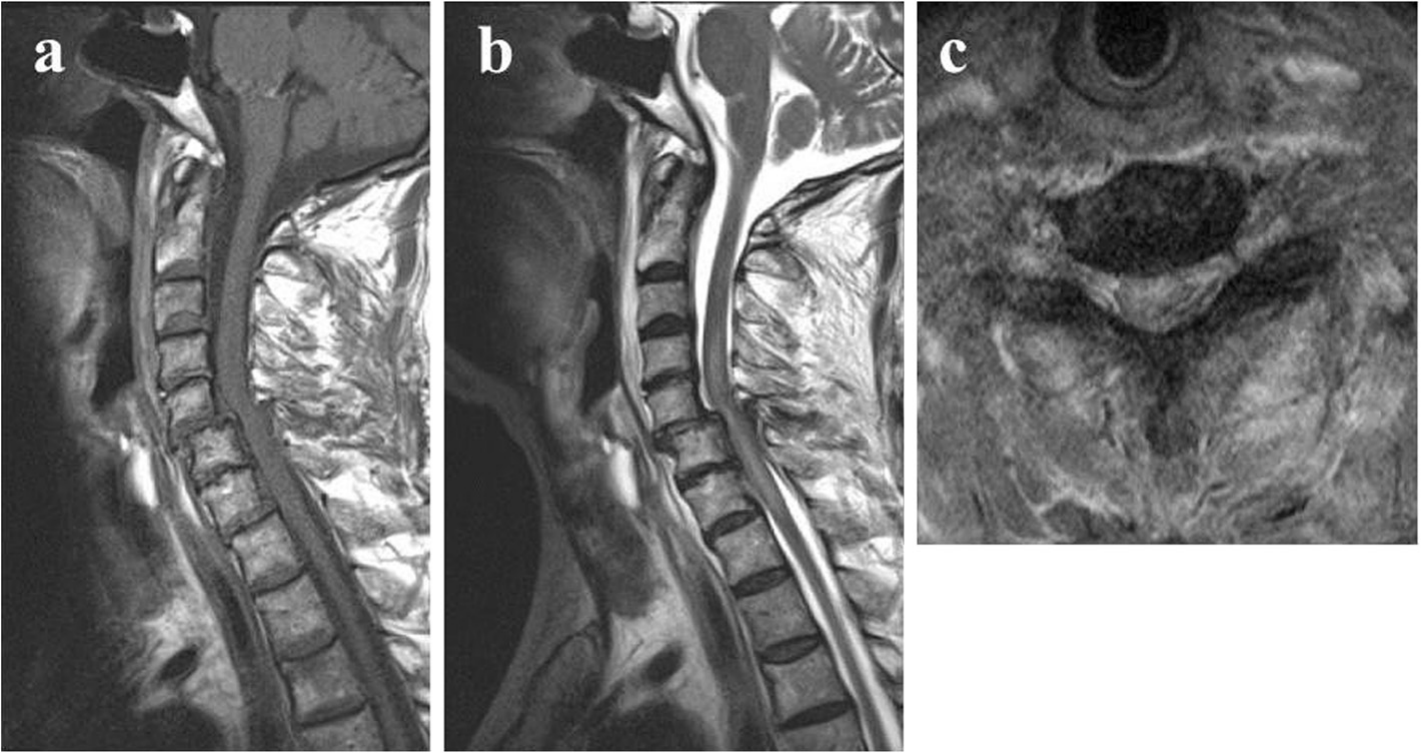
**Magnetic resonance imaging showing spinal canal stenosis and swelling of the spinal cord eight weeks after the traffic accident. (a)** T1-weighted sagittal image; **(b)** T2-weighted sagittal image; **(c)** T2-weighted axial image.

**Figure 3:**
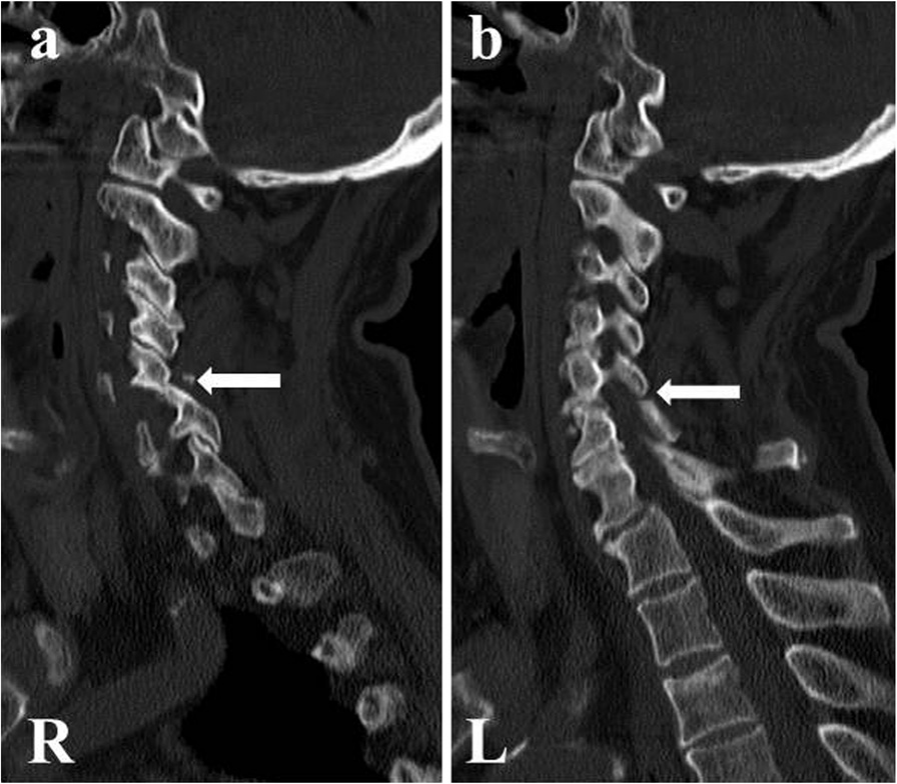
**Sagittal computed tomography performed eight weeks after the traffic accident showing dislocation of both facet joints at C5 to C6. (a)** R: right side (arrow). **(b)** L: left side (arrow).

We carried out an X-ray image examination of our patient’s cervical spine and diagnosed a slight cervical spondylolisthesis (Figure 1a). CT and MRI were not performed. Because our patient did not show motor weakness or paralysis, his neck and arm pain were treated conservatively. However, the pain did not change over the six weeks following the injury. We conducted further X-ray imaging, MRI and CT eight weeks after the injury (Figures 1a,b,c, 2 and 3). Plain X-ray film images obtained at this time showed increased instability at the C5 to C6 joint when compared with those taken immediately after the accident (Figure 1a,b,c). MRI revealed central spinal canal stenosis at the C5 to C6 joint and high signal intensity in the spinal cord on T2-weighted imaging (Figure 2). A sagittal CT showed bilateral dislocation of facet joints (Figure 3).

We planned posterior-anterior surgery eight weeks after the injury. We performed a partial resection of both C5 to C6 facet joints for reduction, using a posterior approach. Half of the C5 to C6 facet joint was resected on the right side, and one quarter of the C5 to C6 facet joint was resected on the left side.

Lateral mass screws on the left side were used for fixation, and bilateral local bone was grafted between the posterior surface of the C5 and C6 laminae. Lateral mass screws on the right side were not used for fixation because most of the lateral bone mass was resected. Because fixation was insufficient, we added an anterior approach by removing the C5 to C6 intervertebral disc, and grafted iliac bone into the C5 to C6 space. We used a titanium plate for fixation.

**Figure 4:**
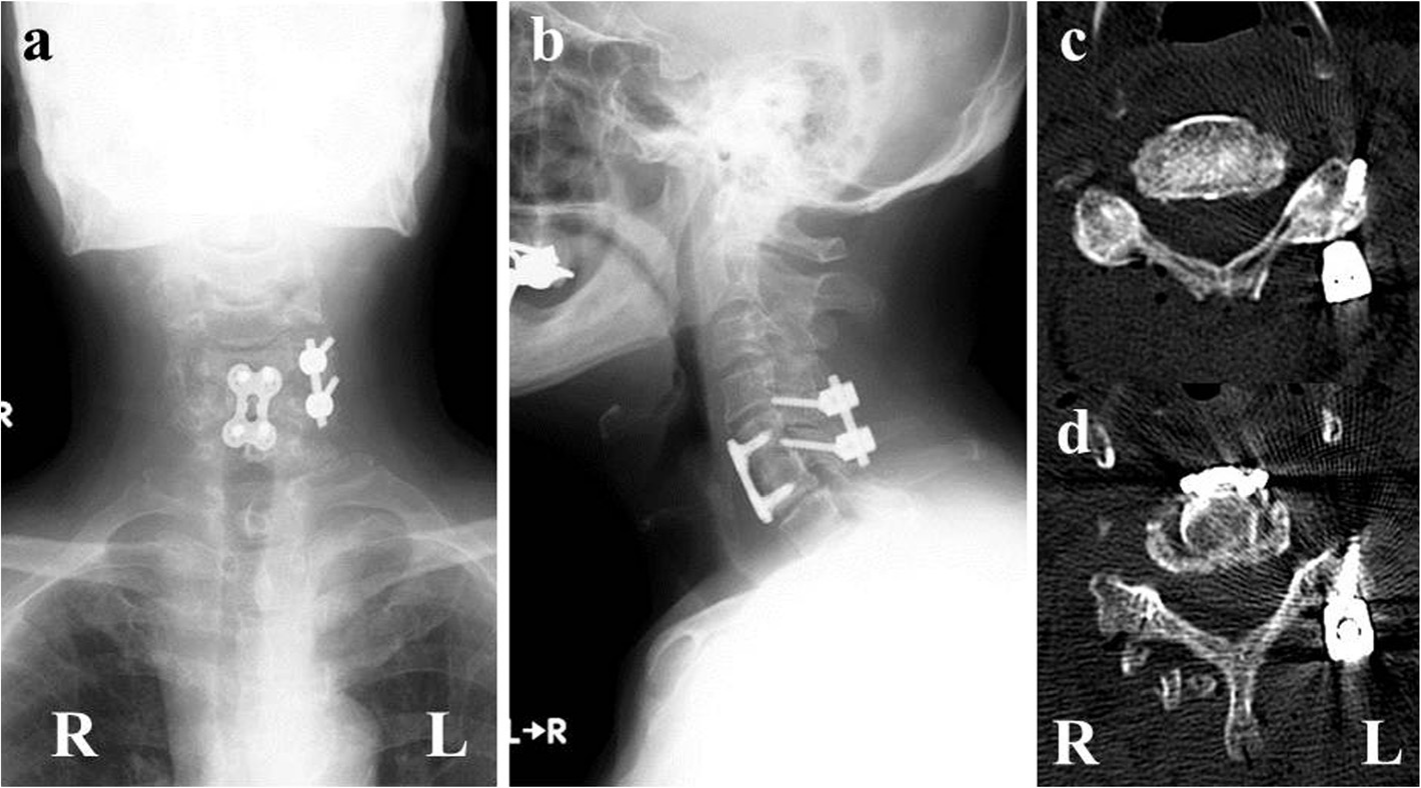
**Plain X-ray imaging and computed tomography scans obtained six months after surgery.** We performed surgery to remove a part of both sides of the facet joints at the C5 to C6 joint, and posterior fusion with lateral mass screws on the left side. After posterior fusion, anterior fusion using a plate and screws was performed. **(a)** X-ray anterior-posterior view; **(b)** X-ray lateral view; **(c)** computed tomography scan of C5; **(d)** computed tomography scan of C6. R: right side. L: left side.

**Figure 5:**
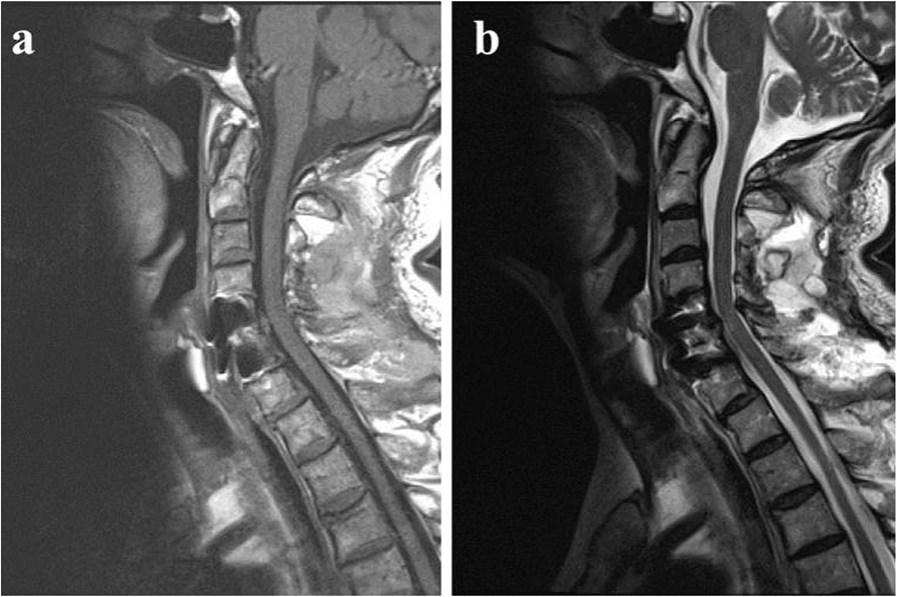
**Magnetic resonance imaging three months after surgery.** Space in the spinal canal has increased, and swelling of the spinal cord has decreased three months after surgery. **(a)** T1-weighted image; **(b)** T2-weighted image.

Our patient gradually became symptom-free after surgery. He presented with a transient C5 palsy on his right side; however, he had recovered three months after the surgery. Plain X-ray film images obtained six months after surgery showed good stability (Figure 4). MRI revealed recovery of the central spinal canal stenosis at the C5 to C6 joint and showed normal intensity in the spinal cord on T2-weighted imaging (Figures 2 and 5).

## Discussion

We present the case of an old bilateral cervical facet joint dislocation fracture found in a 76-year-old man, treated by posterior-anterior reduction and fixation. After surgery, bony union was achieved, and our patient’s neck pain and arm pain disappeared.

In one study, cervical spine injuries were identified in 740 patients and the diagnosis was delayed or missed in 34 patients (4.6%). Ten of the 34 patients (29%) developed permanent sequelae as a result of these delays [1]. In another study, 1331 patients were evaluated following blunt trauma injury; recognition of their cervical spine injury was delayed in 8% of patients [2]. In a review of 253 patients with 274 spinal injuries, delays in diagnosis were documented in 22.9% of cervical injuries and 4.9% of thoracolumbar injuries [3]. We therefore carefully consider the potential misdiagnosis of cervical injury before therapy. In our patient, even with slight cervical spondylolisthesis, plain X-rays were not sufficient for a proper diagnosis. In general, such a finding requires further diagnostic efforts including a CT scan.

Currently, there is evidence in several articles that closed reduction in an awake and alert patient is relatively safe [14,15]. There is, however, no agreement regarding whether such a maneuver is safe in an obtunded or intubated patient. Most surgeons would agree that an open reduction with or without a decompression should be considered in patients where there is MRI evidence of a failed closed reduction or what is considered to be a dangerous herniated disc before reduction [14]. We had attempted closed reduction while our patient was awake, but this failed. We therefore selected open reduction.

A few authors have reported surgical treatment for old dislocations of the subaxial cervical spine, including bilateral facet joint dislocation. The fusion rate and improvement in symptoms after surgery were excellent in all but one of 16 surgically treated patients who had sustained an old injury of their lower cervical spine, including dislocation [13]. Reduction and fusion surgery was performed in 12 patients with an old dislocation of the lower cervical spine, including bilateral dislocation of the facet joints, and all patients developed bone fusion and showed neurological improvement [9]. There has been a report of three patients who had older (>8 weeks) untreated bilateral cervical facet dislocation. All three patients were surgically treated [10]. In a neurologically intact 51-year-old patient, surgical treatment was performed 10 weeks after the trauma upon diagnosis of bilateral cervical facet dislocation at the C5 to C6 joint. That patient remained neurologically intact and radiographic fusion was observed [11]. In our case, our patient with an old bilateral cervical facet joint dislocation fracture underwent surgery; complete reduction was performed, and bony fusion was observed. We therefore recommend reduction and fixation surgery if the patient has an old bilateral cervical facet joint dislocation fracture.

Several authors have reported surgical methods for patients with old bilateral cervical facet joint dislocation fractures using anterior and posterior approaches, and a combination of the two with instrumentation [9-11]. The use of pedicle screws for patients with old bilateral cervical facet joint dislocation fractures has not been frequently reported. Yamazaki *et al*. reported a case of an old fracture-dislocation unilaterally at a C4 to C5 facet joint. Reduction of the deformity and spinal fusion were successfully performed using a pedicle screw-rod system [12]. Lee *et al*. reported that, when patients were neurologically intact, an anterior approach was more commonly chosen than a posterior approach, and combined approaches were more commonly chosen for bilateral facet injuries [16]. In our case, we selected a bilateral posterior facetectomy, unilateral lateral mass screws and anterior plating for our patient. To reduce the facet joint, half of the C5 to C6 facet joint was resected on the right side, and one quarter of the C5 to C6 facet joint was resected on the left. We considered an anterior plate to be insufficient for stabilization. Furthermore, half of the C5 to C6 facet joint was resected, so we could not use lateral mass screws on the right side.

Several surgical methods are recommended for old bilateral cervical facet dislocation fractures. We recommend these methods be further investigated to identify those that achieve good reduction and fixation without complications.

## Conclusion

We have reported a case of an old bilateral cervical facet joint dislocation fracture found in a 76-year-old man, treated by posterior-anterior reduction and fixation. After surgery, his neck pain and arm pain disappeared and bony union was achieved. We recommend reduction and fixation surgery for patients with an old dislocation fracture of the cervical spine.

## Consent

Written informed consent was obtained from the patient for publication of this case report and accompanying images. A copy of the written consent is available for review by the Editor-in-Chief of this journal. The protocols for human procedures used in this study were approved by the ethics committee of our institution.

## Competing interests

The authors declare that they have no competing interests.

## Authors’ contributions

SO, TS and GI performed the surgery. HSs and KT evaluated the imaging. The other authors were key contributors in writing the manuscript. All authors read and approved the final manuscript.
